# Majority of Chinese Medicine Herb Category “Qing Re Yao” Have Multiple Mechanisms of Anti-inflammatory Activity

**DOI:** 10.1038/s41598-018-25813-x

**Published:** 2018-05-09

**Authors:** Fulan Guan, Wing Lam, Rong Hu, Yun Kyung Kim, Hua Han, Yung-Chi Cheng

**Affiliations:** Department of Pharmacology, Yale University School of Medicine, New Haven, Connecticut 06510 USA

## Abstract

Herbs categorized as “Qing Re Yao” are translated into “medicine that removes heat” where heat symptoms strongly resemble inflammation. 226 herbs, among those 54 herbs are classified as “Qing Re Yao”, were studied on six key mechanisms of inflammation: COX2, iNOS activity, and the pathways of IL-6, IFNγ, TNF-α and glucocorticoid in order to assess if the majority of this family of herbs have anti-inflammatory activity. 96% demonstrated at least one anti-inflammatory process or innate immunity modular activity, and 72% could affect one anti-inflammatory process. Of the, 54 “Qing Re Yao” 68% affect at least 2 mechanism compared to only 4% (47 herbs) in the “Bu Yi Yao” category that are used to “tonify body energy” and prevent diseases. Moreover 43% of “Qing Re Yao” herbs affect 3 or more mechanisms while none of the “Bu Yi Yao” have this poly-mechanism quality. Additionally “Qing Re Yao” herbs exhibiting activity against STAT3 or GAS could have downstream effects on these target genes and their pathways. Our study addresses the key action on why “Qing Re Yao” work on inflammation. This study also demonstrates the utility in isolating anti-inflammatory substances to be used as a lead for drug discovery and development.

## Introduction

For over 2000 years, the important role Traditional Chinese medicine (TCM) has played in the healthcare of Asia has not been accepted globally because a lack of scientific evidence supports its claims. TCM herbs are classified into several categories based on their usage. One major category, “Qing Re Yao”, is used for the treatment of “Re” syndrome. In Mandarin the words “Qing” means “remove”, “Re” means “heat” and “Yao” means “medicine”. From the perspective of Chinese medicine “Qing Re Yao” translates into “medicines that treat heat”. According to the observations of Chinese medical practitioners, diseases with “heat” syndrome are apparently associated with different types of inflammation related symptoms. Consequently, it is thought “Qing Re Yao” can be used to treat different groupings of inflammatory associated diseases. Some studies on individual herbs and their anti-inflammatory properties have already been done by others for but there has never been a study done on the entire category.

Inflammation is a natural physiological response to pathogen infections, foreign substances, and pro-inflammatory substances. It is triggered by the pathological state of tissue as well as the aging process. Cytokine and hormone systems play an important role in inflammatory processes and manifest in the form of fever, allergy, etc. The inhibition of cyclooxygenase-2 (COX2) and Inducible nitric oxide synthase (iNO)S enzyme activity, the signaling pathways of Interleukin 6 (IL-6)-STAT3, Tumor necrosis factor alpha (TNF-α) -NFκB and Interferon gamma (IFNγ) -GAS activity as well as the stimulation of Dexamethasone (Dexa) -GRE activity are all involved in anti-inflammatory response^1–16^.

Toll Like receptors (TLR) act as innate immune sensors that recognize microbial pathogen-associated molecular patterns and endogenous “danger” molecules which are released from host cells as a consequence of infection, inflammation, cell death or stress^17,18^. Their activities could modulate the immune-function of individuals. In this study we also examined it.

Another category of herbs “Bu Yi Yao” was studied. “Bu” means supplement and “Yi” means benefit. The translation equates to “medicine that benefits health”. These herbs are often used to treat those with ill health and aging-associated disease. The two categories were compared in order to assess how the “remove heat” category was related to anti-inflammation activity compared with another classes and to investigate the scientific basis behind it.

For the first time our study provides solid scientific evidence that explains how the majority of the “Qing Re Yao” herbs can affect multiple anti-inflammatory pathways. Most importantly this study attempts to address the lack of scientific evidence in order to support more widespread usage of TCM in western medicine.

## Result

### The mechanism spectrum of Chinese medicine herbs that affect inflammatory processes and the innate immunity pathway

According to the observations of Chinese medical practitioners most diseases were associated with “heat” syndrome. In order to assess whether Chinese herbs that were used frequently in clinics have anti-inflammatory activity or immunity modulatory activity, the impact of 226 TCM herbs, (54 of which are classified as “Qing Re Yao”), on the expression of different pathway genes and luciferase at various concentrations (28, 83, 250, 750 μg/ml) was examined on different signal pathways or up to 4 mg/ml herb on iNOS and COX2 enz activity. Dose response data was analyzed. (Data was not shown) Inhibition was defined as more than 50% inhibition and stimulation was defined as more than three times increase over the baseline of stimulator activity at less than 750 µg/ml or 4 mg/ml respectively. For herbs with no activity against inflammatory activity, we tested different batches of the same herbs in order to ensure that its lack of activity was not due to being inert.

**Figure 1:**
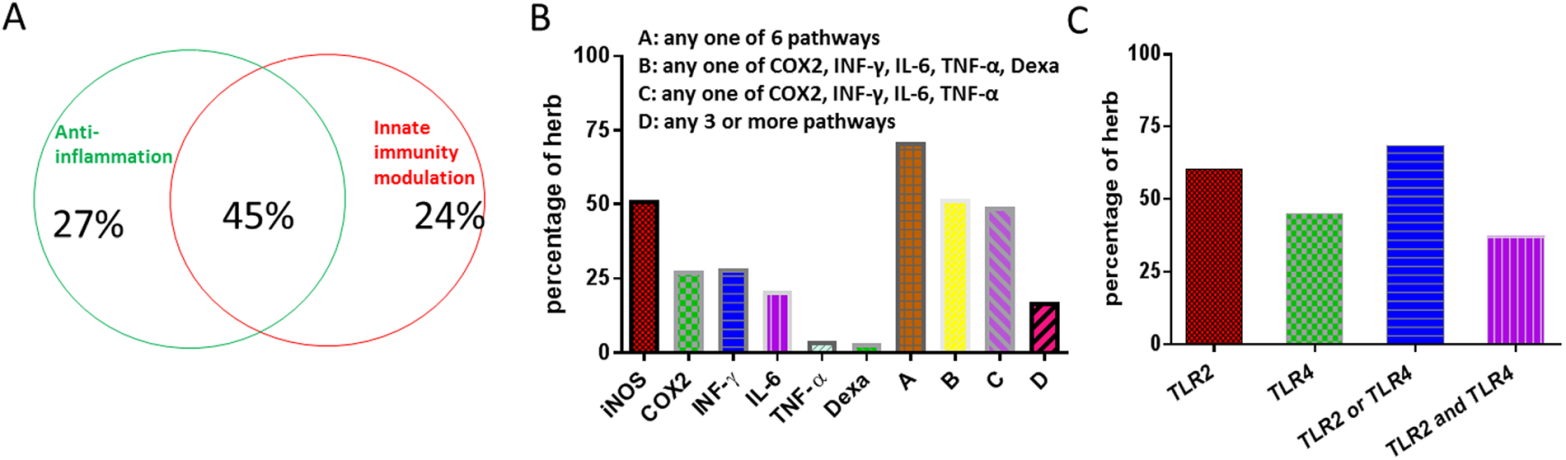
Anti-inflammatory action and stimulation of innate immunity of Chinese herb medicine (226). (**A**) Spectrum of all herbs in affecting 6 inflammatory processes and 2 innate immunity pathways. (**B**) Percentage of all herbs in affecting 6 inflammatory processes. (**C**) Percentage of all herbs in affecting 2 innate immunity pathways.

As shown in the results of Fig. 1, Most of the Chinese medicine herbs had anti-inflammatory and/or innate immune-modulating activity. 96% of the herbs could have anti-inflammatory process or innate immuno-modulatory activity (Fig. 1A); 72% of herbs could affect inflammatory processes (Fig. 1A,B); 69% of the herbs could stimulate the TLR2 or TLR4 pathway; and 45% of herbs could have both anti-inflammatory activity and innate immunity (Fig. 1A,C).

Of the inflammatory pathways studied the most common is iNOS inhibition while glucocorticoid pathway activation is the least common. Also 69% demonstrated either TLR2 or TLR4 response. This supports the notion that Chinese medicine has innate immunity modulating activity.

### The effect of “Qing Re Yao” and “Bu Yi Yao” on enzyme and signaling pathway activity

**Figure 2:**
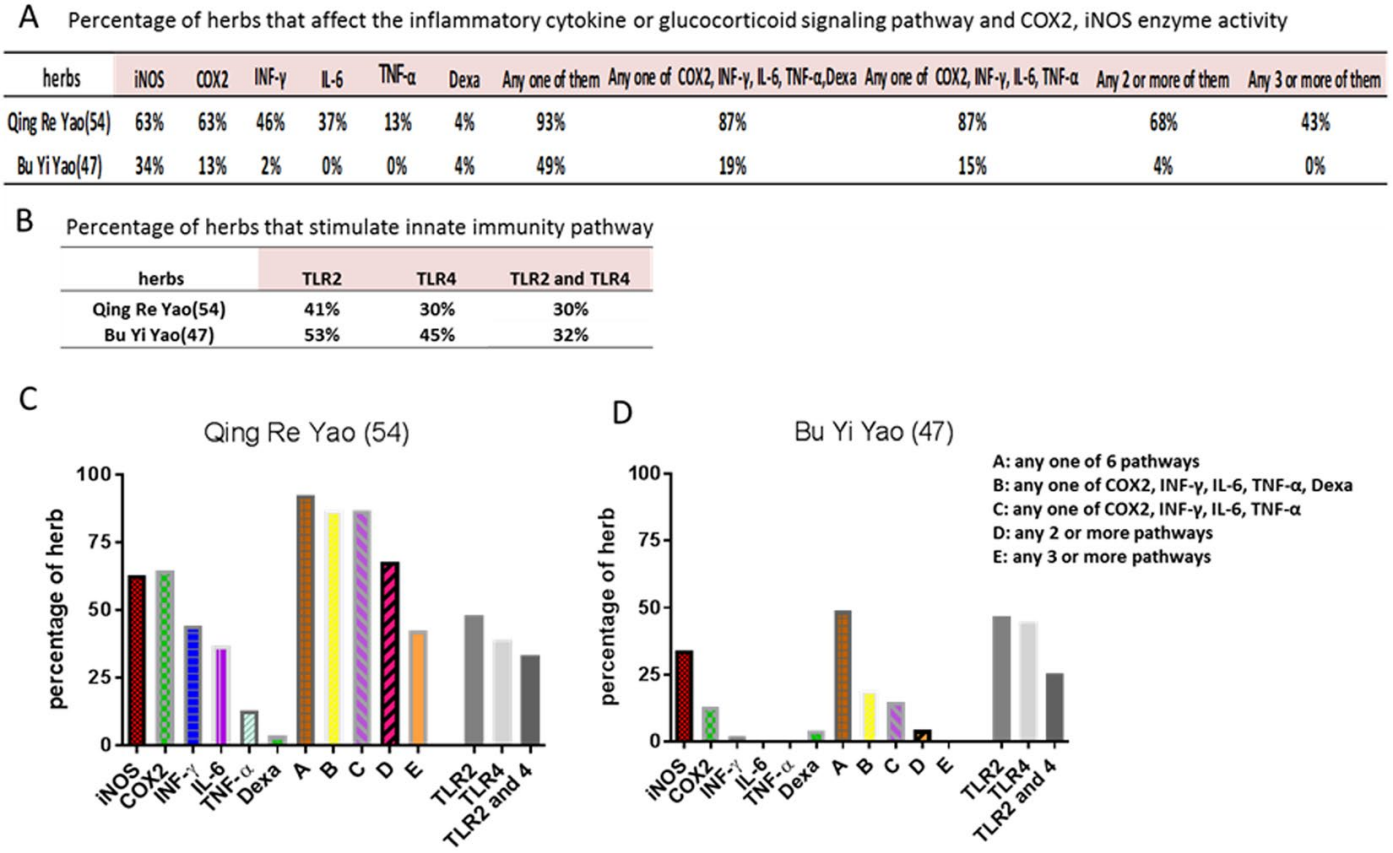
Anti-inflammatory action and stimulation of innate immunity of two categories of Chinese herb medicine. (**A**) Percentage of “Qing Re Yao” and “Bu Yi Yao” in affecting on the inflammatory cytokine or glucocorticoid signaling pathway and COX2, iNOS enzyme activity. (**B**) Percentage of “Qing Re Yao” and “Bu Yi Yao” in affecting on innate immunity pathway. (**C**) Percentage of “Qing Re Yao” in affecting on inflammatory processes and innate immunity pathways. (**D**) Percentage of “Bu Yi Yao” in affecting on inflammatory processes and innate immunity pathways. Inhibition was cut defined as more than 50% inhibition; stimulation was defined more than 3 times stimulated at concentration of herbs under 750 µg/ml in luciferase reporter signaling assay or under 4 mg/ml in enzyme assay.

TCM herbs are classified into several classes based on usage. “Qing Re Yao” is a major category used to treat “heat” diseases that resemble inflammation symptoms. Another major category “Bu Yi Yao” is used for the tonification of body ailments. These two categories of Chinese herbs were chosen to assess if “Qing Re Yao’s” usage purpose relates to anti-inflammatory activity compared with Bu Yi Yao, which is not prescribed for the same types of disease. Furthermore we investigated whether there is scientific evidence to prove that “Qing Re Yao” affects anti-inflammatory activity. The responses to different dosages of herbs (28, 83, 250, 750 μg/ml) with and without *E.coli* β-glucuronidase treatment were examined for its impact on luciferase expression on different signal pathways or up to 4 mg/ml herb on iNOS and COX2 enzyme activity. The results for “Qing Re Yao” are shown in Table 1 and Fig. 2A: 63% (34/54) inhibited COX2 activity, 63% (34/54) inhibited iNOS activity, 46% (25/54) inhibited the IFNγ-GAS signaling pathway, 37% (20/54) inhibited IL-6-STAT3 signaling pathway, 13% (7/54) inhibited the TNF-α-NFκB signaling pathway, and 4% (2/54) stimulated the Dexamethasone- GRE signaling pathway. We defined if the herb has inhibition or stimulation activity when it showed inhibition or stimulation activity without β-glucuronidase treatment. Moreover 93% of “Qing Re Yao” affected at least one signaling pathway or enzyme activity while only 49% of “Bu Yi Yao” affected at least one pathway or enzyme activity; 68% of “Qing Re Yao” affect at least two mechanisms compared to 4% of “Bu Yi Yao”; 43% of “Qing Re Yao” herbs affect 3 or more mechanisms while none of the Bu Yi Yao have this poly-mechanism quality (Fig. 2A,C,D). Compared to “Bu Yi Yao”, iNOS inhibition was not unique and the inhibition of INF-γ, IL-6, TNF-α and COX2 were the most common for “Qing Re Yao” (Fig. 2, Tables 1, 2). It can be concluded that the majority of “Qing Re Yao” herbs demonstrated activity toward more than one mechanism related to inflammation. It was also noted that different “Qing Re Yao” herbs had a unique spectrum of activity on different anti-inflammatory mechanisms (Table 1): i.e herb 20 affected 5 mechanisms, whereas herb 5, 10, 19, 28, 29, 48 affected 4 mechanisms, while others affected fewer mechanisms. Every herb had its unique anti-inflammatory properties. For example, many herbs demonstrated a biphasic effect where some herbs stimulated the pathway under low concentrations while inhibiting the same pathway under high concentrations (herb 10, 24 and 35). This could help explain why the same herbs are used differently in separate TCM formulas.

**Table 1:** Effect of Qing Re Yao on enzyme activity for proinflammatory chemical synthesis or inflammatory cytokine or glucocorticoid signaling pathways.

Item No.	TNF-α	IL-6	INF-γ	Dexa	COX2	iNOS	Item No.	TNF-α	IL-6	INF-γ	Dexa	COX2	iNOS
Qing Re Xie Huo (9)							Qing Re Jie Du (27)						
1	—	**↑**	↓	—	↓	↓	28	↓	—	↓	—	↓	↓
2	—	—	—	—	↓	↓	29	↓	↓	↓	↓	↓	—
3	—	↓	—	—	—	↓	30	—	↓	↓	—	↓	↓
4	—	—	—	—	—	—	31	—	—	—	—	↓	↓
5	—	↓	↓	—	↓	↓	32	—	—	—	—	↓	—
6	—	—	—	—	↓	—	33	—	—	—	—	—	↓
7	—	—	—	—	↓	—	34	—	—	—	—	↓	↓
8	—	↓	↓	↓	↓	↓	35	—	**↑**↓	—	—	↓	↓
9	—	—	—	—	—	↓	36	—	—	↓	**↑**	—	↓
Qing Re Zhao Shi (7)							37	—	↓	↓	—	—	↓
10	↓	**↑**↓	↓	↓	↓	—	38	—	—	—	—	↓	—
11	—	↓	↓	—	↓	—	39	—	—	↓	—	—	↓
12	—	↓	—	—	—	↓	40	—	—	↓	—	↓	↓
13	—	—	—	—	—	—	41	—	—	↓	—	—	↓
14	—	↓	—	—	↓	↓	42	—	—	↓	—	↓	↓
15	—	—	—	—	—	—	43	—	**↑**	—	—	↓	—
16	—	—	—	—	↓	—	44	—	—	—	—	—	↓
Qing Re Liang Xue (5)							45	—	—	↓	—	—	—
17	—	—	—	—	↓	↓	46	—	↓	—	—	—	↓
18	—	—	↓	—	—	↓	47	—	—	—	—	↓	↓
19	↓	↓	↓	↓	—	↓	48	↓	↓	↓	↓	↓	—
20	↓	↓	↓	↓	↓	↓	49	—	—	—	—	—	—
21	—	—	—	↓	↓	↓	50	—	—	—	—	↓	—
Qing Xu Re (6)							51	—	↓	↓	—	—	↓
22	—	—	↓	—	↓	—	52	—	—	—	—	↓	↓
23	↓	↓	↓	↓	—	—	53	—	—	—	—	↓	—
24	—	**↑**↓	—	—	↓	↓	54	—	↓	↓	—	↓	↓
25	—	—	—	**↑**	↓	—							
26	—	—	↓	—	↓	↓							
27	—	↓	↓	—	—	↓							

TNF-α: TNF-α-NFκB luciferase reporter; IL-6: IL-6-STAT3 luciferase reporter; INF-γ: INFγ-GAS luciferase reporter; Dexa: Dexamethasone-GRE luciferase reporter; COX2: COX2 enzyme activity; iNOS: iNOS enzyme activity. ↓: inhibition ≥ 50%; **↑**: stumulation ≥ 3 folds; —: no impact w/o β-glucuronidase treatment at concentration ≤ 750 µg/ml in the inflammatory cytokine or glucocorticoid signaling pathway and at concentration ≤ 4 mg/ml in COX2, iNOS enzyme activity assay.

**Table 2:** Effect of Bu Yi Yao enzyme activity for proinflammatory chemical synthesis or inflammatory cytokine or hormone signaling pathways.

Item No	TNF-α	IL-6	INF-γ	Dexa	COX2	iNOS	Item No	TNF-α	IL-6	INF-γ	Dexa	COX2	iNOS
55	—	—	—	—	—	↓	79	—	—	**↑**	**↑**	—	—
56	—	—	—	—	—	—	80	—	—	—	—	—	—
57	—	—	—	—	—	—	81	—	—	—	—	—	—
58	—	—	—	—	—	—	82	—	**↑**	**↑**	—	—	↓
59	—	—	—	—	—	—	83	—	—	**↑**	—	—	—
60	—	**↑**	—	—	—	—	84	—	—	—	—	—	—
61	—	—	—	—	—	↓	85	—	—	—	—	—	—
62	—	—	—	—	—	↓	86	—	—	—	—	—	↓
63	—	—	—	—	—	—	87	—	—	—	—	—	—
64	—	—	—	—	↓	—	88	—	—	—	—	—	—
65	—	—	—	—	↓	↓	89	—	—	—	—	—	↓
66	—	**↑**	—	—	—	↓	90	—	—	—	—	↓	—
67	—	—	—	—	—	↓	91	—	—	—	—	—	↓
68	—	—	—	—	—	—	92	—	—	—	—	—	—
69	—	—	—	↓	—	↓	93	—	—	—	—	—	↓
70	—	—	—	—	↓	—	94	—	—	—	—	—	—
71	—	—	—	—	—	—	95	—	**↑**	↓	—	—	—
72	—	—	—	—	—	—	96	—	**↑**	—	↓	—	↓
73	—	—	—	—	—	↓	97	—	**↑**	—	—	—	—
74	—	—	—	—	—	↓	98	—	—	—	—	↓	—
75	—	—	—	—	—	—	99	—	**↑**	—	**↑**	—	—
76	—	—	—	—	—	—	100	—	—	—	—	—	—
77	—	—	—	—	—	—	101	—	—	—	—	—	—
78	—	—	—	—	↓	↓							

TNF-α: TNFα-NFκB luciferase reporter; IL-6: IL-6-STAT3 luciferase reporter; INF-γ: INFγ-GAS luciferase reporter; Dexa: Dexamethasone-GRE luciferase reporter; COX2: COX2 enzyme activity; iNOS: iNOS enzyme activity. ↓: inhibition ≥ 50%; **↑**: stimulation ≥ 3 folds; —: no impact w/o β-glucuronidase treatment at concentration ≤ 750 µg/ml in the inflammatory cytokine or glucocorticoid signaling pathway and at concentration ≤ 4 mg/ml in COX2, iNOS enzyme activity assay.

In regards to the different classes of “Qing Re Yao”: Qing Re Xie Huo Yao, Qing Re Zao Shi Yao, Qing Re Jie Du Yao, Qing Re Liang Xue Yao, and Qing Xu Re Yao, attempts were made to assess whether the different classes could be associated with different mechanisms of inflammation. We observed no apparent differences among the 5 different classes of “Qing Re Yao” and the results are deprecated on Table 1, 89% (8/9) of Qing Re Xie Huo Yao, 57% (5/7) of Qing Re Zao Shi Yao, 100% (5/5) of Qing Re Liang Xue Yao, 96% (26/27) of Qing Re Jie Du Yao, 100% (6/6) of Qing Xu Re Yao could affect any one of the above inflammatory signaling pathways or enzyme activities in different combinations (Table 1). In order to adequately address the question, further study will be needed using more species of herbs from each class.

Because of the critical role TLR2 and TLR4 pathways play in modulating innate immunity, we also assessed the activity of “Qing Re Yao” and “Bu Yi Yao” on each pathway. For “Qing Re Yao”: 41% (22/54) stimulated the TLR2 signaling pathway and 30% (16/54) stimulated the TLR4 signaling pathway. For “Bu Yi Yao”: 53% (25/47) stimulated the TLR2 signaling pathway and 45% (21/47) stimulated the TLR4 signaling pathway. There were no apparent differences between “Qing Re Yao” and “Bu Yi Yao”. Table 3, Fig. 2B shows that both “Qing Re Yao” and “Bu Yi Yao” can modulate innate immunity through the TLR2 and TLR4 receptor to almost the same degree irrespective of category.

**Table 3:** Effect of Qing Re Yao and Bu Yi Yao on TLR2 and TLR4 signaling pathways.

0	TLR2	TLR4	Item No	TLR2	TLR4	Item No	TLR2	TLR4	Item No	TLR2	TLR4
1	—	—	28	—	—	55	—	—	79	—	—
2	—	—	29	—	**↑**	56	—	—	80	**↑**	**↑**
3	**↑**	—	30	**↑**	—	57	—	**↑**	81	**↑**	**↑**
4	—	—	31	—	—	58	—	—	82	**↑**	**↑**
5	—	—	32	**↑**	**↑**	59	**↑**	—	83	**↑**	—
6	**↑**	—	33	—	—	60	—	**↑**	84	—	—
7	—	—	34	**↑**	**↑**	61	—	—	85	—	—
8	—	—	35	—	—	62	—	—	86	—	—
9	**↑**	**↑**	36	**↑**	—	63	**↑**	—	87	—	**↑**
10	—	—	37	**↑**	**↑**	64	**↑**	**↑**	88	—	**↑**
11	—	—	38	**↑**	**↑**	65	**↑**	**↑**	89	—	**↑**
12	**↑**	**↑**	39	**↑**	**↑**	66	—	—	90	**↑**	—
13	—	—	40	**↑**	—	67	**↑**	—	91	**↑**	**↑**
14	—	**↑**	41	**↑**	—	68	—	**↑**	92	**↑**	**↑**
15	**↑**	**↑**	42	**↑**	**↑**	69	**↑**	**↑**	93	—	—
16	**↑**	**↑**	43	—	—	70	**↑**	**↑**	94	**↑**	—
17	—	—	44	**↑**	**↑**	71	**↑**	**↑**	95	—	—
18	—	—	45	**↑**	**↑**	72	**↑**	—	96	—	—
19	**↑**	—	46	**↑**	—	73	**↑**		97	**↑**	—
20	—	**↑**	47	—	—	74	**↑**	**↑**	98	—	—
21	—	—	48	—	—	75	**↑**	**↑**	99	**↑**	**↑**
22	**↑**	**↑**	49	**↑**	**↑**	76	—	—	100	**↑**	**↑**
23	—	—	50	**↑**	**↑**	77	**↑**	—	101	—	—
24	**↑**	**↑**	51	**↑**	**↑**	78	**↑**	**↑**			
25	—	—	52	—	—						
26	—	—	53	—	—						
27	—	—	54	**↑**	**↑**						

TLR2: PGN-TLR2 luciferase reporter; TLR4: LPS-TLR4 luiferase reporter. ↓: inhibition ≥ 50%; **↑**: stimulation ≥ 3 folds; —: no impact at concentration ≤ 750 µg/ml.

### Different “Qing Re Yao” with the same pathway inhibitory activity could have different downstream target impact

In the reporter assay, different herbs could inhibit the same transduction pathways but still hit different targets. In order to address this, it was necessary to check how the downstream mRNA is further affected. In this study we observed the impact of those herbs on STAT3 and GAS downstream target genes.

STAT3 signaling is a major intrinsic pathway for IL-6 action. It is capable of transcripting a large number of genes that are crucial for inflammation, such as COX2, B-cell lymphoma 6 (BCL6), JunB Proto-Oncogene (JUNB) and Interleukin-23 (IL23)^19,20^.

**Figure 3:**
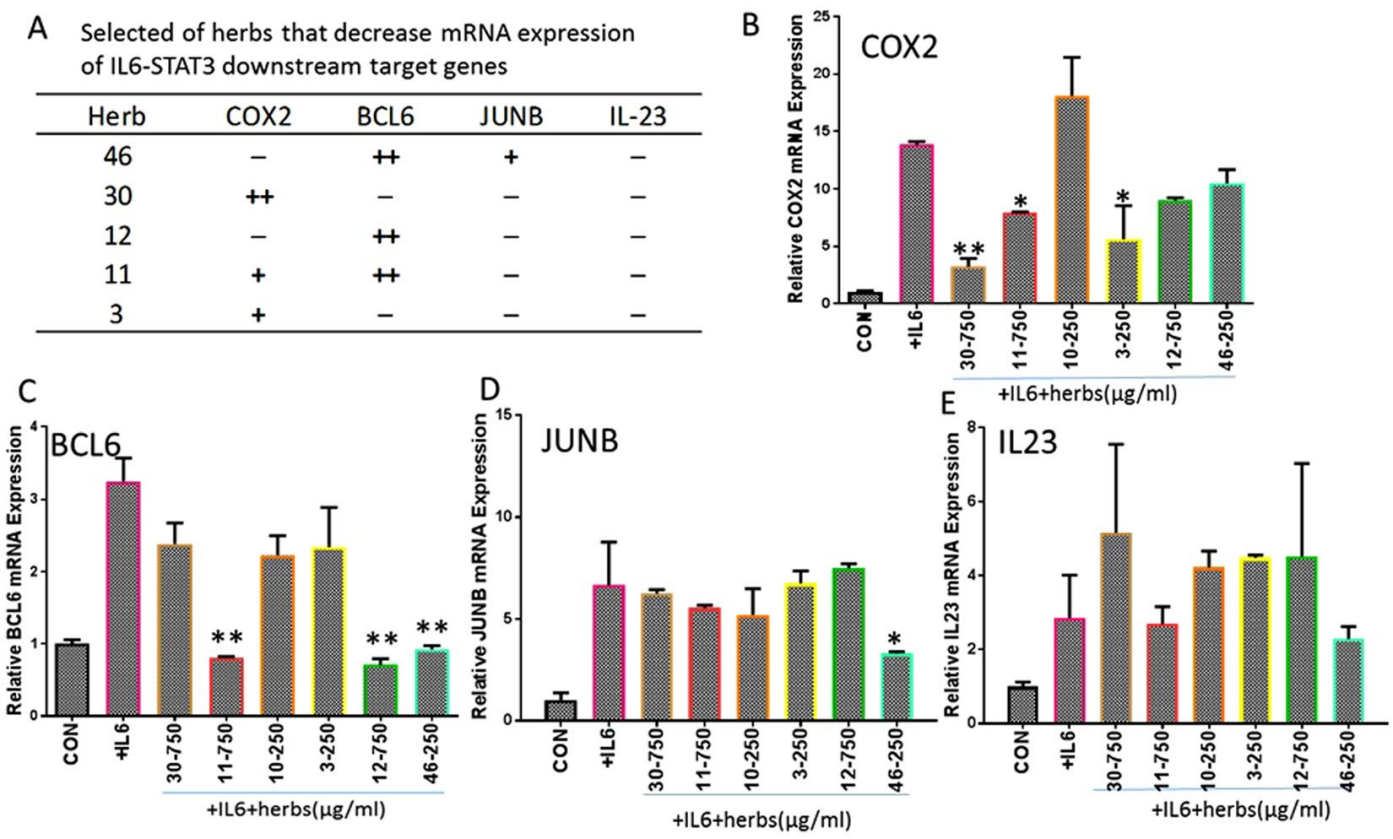
Impact of selected herbs on different IL-6-STAT3 downstream gene expression. (**A**) Table of herbs inhibited IL-6 induced mRNA expression of COX2, BCL6, JUNB and IL23. (**B**–**E**) Quantitative real time PCR for mRNA expression of STAT3 target gene COX2, BCL6, JUNB and IL23. Each bar represents a mean of three different experiments (triplicate samples of each). Compare with +IL-6 group, ^+,^*p < 0.05; ^++,^**p < 0.01; −: no impact.

In order to examine the impact on IL-6 induced mRNA expression of COX2, BCL6, JUNB and IL23, we selected 6 kinds of “Qing Re Yao” that showed significant inhibitory activity on the IL-6-STAT3 luciferase reporter assay. The dosage was determined according to the IC_50_ of herbs on inhibition of the IL-6-STAT3 luciferase signal. The result revealed a unique impact spectrum induced on IL-6: herbs 30, 11, 3 could decrease mRNA expression of COX2 (Fig. 3A,B); herbs 11, 12, 46 could decrease mRNA expression of BCL6 (Fig. 3A,C); herb 46 could decrease mRNA expression of JUNB (Fig. 3A,D); and none of them decreased mRNA expression of IL23 (Fig. 3A,E).

**Figure 4:**
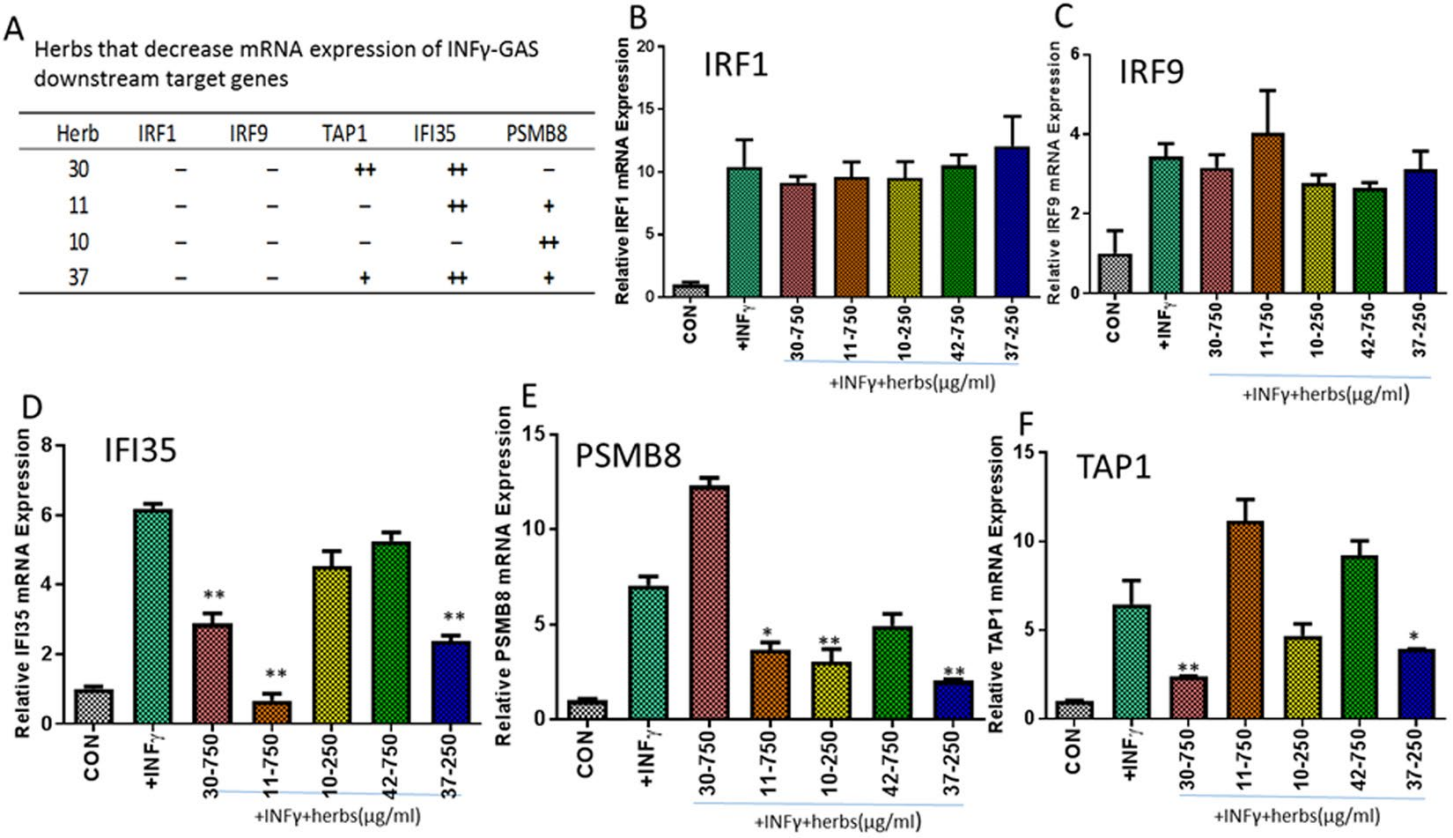
Impact of selected herbs on different INF-γ-GAS downstream gene expression. (**A**) Table of herbs inhibited INFγ induced mRNA expression of IRF1, IRF9, TAP1, PSMB8 and IFI35. (**B**–**F**) Quantitative real time PCR for mRNA expression of GAS garget gene IRF1, IRF9, IFI35, PSMB8 and TAP1. Each bar represents a mean of three different experiments (triplicate samples of each). Compare with +INFγ group, ^+,^*p < 0.05; ^++,^**p < 0.01; −: no impact.

In addition to the IL-6-STAT3 pathway, we also examined five “Qing Re Yao” herbs that had inhibitory activity on the IFNγ-GAS pathway (Table 1). These would have different impact on different IFNγ induced GAS target genes, Interferon regulatory factor 1 (IRF1), interferon regulatory factor 9 (IRF9), Transporter associated with antigen processing 1 (TAP1), IFI35 (Interferon induced protein 35) and Proteasome subunit beta 8 (PSMB8) mRNA expression. As shown in the Fig. 4A–F, herbs 30 and 37 but not herb 11 and 10 decreased the mRNA expression of TAP1 (Fig. 4A,F); herbs 30, 11 and 37 but not herb 10 decreased the mRNA expression of PMSB8 (Fig. 4A,E); herbs 11, 10 and 37 but not herb 30 decreased the mRNA expression of IFI35 (Fig. 4A,D). None of the herbs decreased mRNA expression of IRF1 and IRF9 (Fig. 4A–C), moreover herb 42 did not significantly decrease the mRNA expression of any one of those genes (Fig. 4B–F).

**Figure 5:**
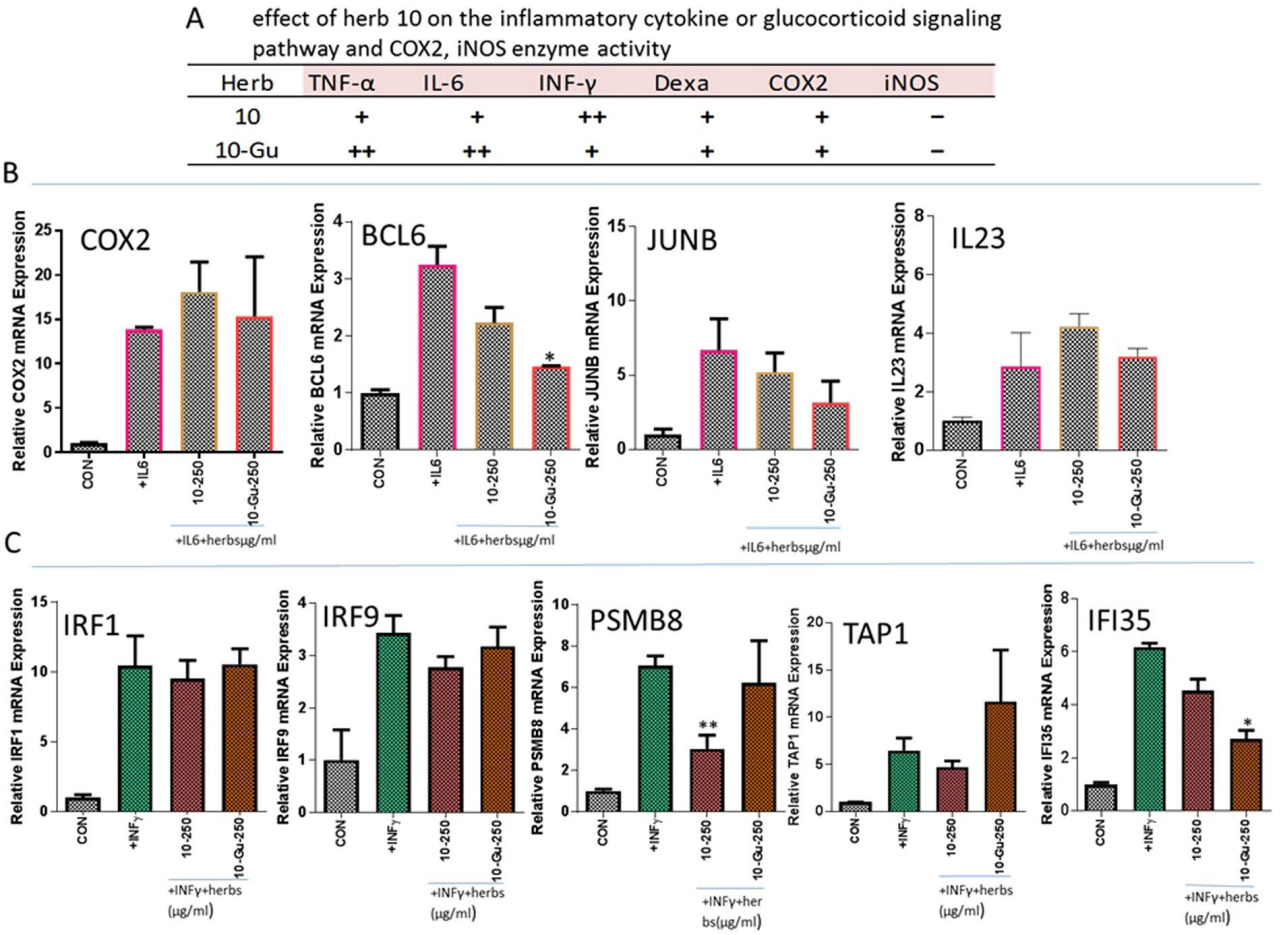
Effect of herb 10 before and after β-glucuronidase treatment. 10-Gu: herb 10 after β-glucuronidase treatment. (**A**) Effect of herb 10 before and after β-glucuronidase treatment on the inflammatory cytokine or glucocorticoid signaling pathway and COX2, iNOS enzyme activity, TNF-α: TNF-α-NFκB luciferase reporter; IL-6: IL-6-STAT3 luciferase reporter; INF-γ: INFγ-GAS luciferase reporter; Dexa: Dexamethasone-GRE luciferase reporter; COX2: COX2 enzyme activity; iNOS: iNOS enzyme activity. +,++: inhibition; −: no impact at concentration ≤750 µg/ml in the inflammatory cytokine or glucocorticoid signaling pathway and at concentration ≤4 mg/ml in COX2, iNOS enzyme activity assay. (**B**) Effect of herb 10 before and after β-glucuronidase treatment on IL-6 induced mRNA expression of COX2, BCL6, JUNB and IL23. Each bar represents a mean of three different experiments (triplicate samples of each). Compare with +IL-6 group, *p < 0.05; **p < 0.01. (**C**) Effect of herb 10 before and after β-glucuronidase treatment on INFγ induced mRNA expression of IRF1, IRF9, TAP1, PSMB8 and IFI35. Each bar represents a mean of three different experiments (triplicate samples of each). Compare with +INFγ group, *p < 0.05; **p < 0.01.

In the Figs 3 and 4, the effects of “Qing Re Yao” on STAT3 and GAS gene expression are shown. Herb 10 did not significantly suppress the mRNA expression of BCL6, COX2, JUNB, IL23 and TAP1, IFI35. Herb 10 which was treated with β-glucuronidase (a major enzyme produced by the bacteria in the gastrointestinal tract), decreased the mRNA expression of BCL6, JUNB and IFI35, but still had no impact on IL-23, COX2 and TAP1 mRNA expression. Untreated herb 10 decreased the mRNA expression of PSMB8 and β-glucuronidase treated herb 10 had no effect on the mRNA expression of PSMB8 (Fig. 5). This indicated the importance of how the glucuronide conjugated aglycone moiety of the chemicals could decrease activity and how it could play an important role in the action selectivity on different tissue or cells. The selectivity could be due to different conjugating activity or glucuronidase activity.

## Discussion

Despite its long history, there has never been scientific-based evidence supporting the idea that Qing Re Yao’s traditional usage in treating heat ailments could also mean inflammation. The findings show that the majority of “Qing Re Yao” possess anti-inflammation properties and supports the idea that the Chinese medicine term for “heat”, (or “Re”), is strongly associated with modern medicine’s concept of inflammation. In the last several decades, the scientific understanding behind inflammation has deepened tremendously: Multiple signaling pathways have been discovered and Inflammation’s role has been proven in numerous disease like cancer, arthritis, cardiovascular disease, Alzheimer’s disease, Parkinson’s disease, diabetes II, digestive tract disease, pulmonary disease, atherosclerosis, and osteoarthritis^21,22^. Our findings indicate 93% of “Qing Re Yao” can have anti-inflammatory activity by affecting one of the inflammatory pathways, however only 49% of “Bu Yi Yao” herbs have anti-inflammatory activity by affecting one inflammatory pathway. 68% “Qing Re Yao” affect two or more inflammatory pathways compared to 4% Bu Yi Yao” herbs, and 43% “Qing Re Yao” affected three or more anti-inflammatory pathways while none of the “Bu Yi Yao” showed this quality. (Figure 2) Among the five sub-classes of “Qing Re Yao”, there is no apparent differences among multiple nature of anti-inflammatory activity (Table 1). On a finer level, the different classes making up “Qing Re Yao” may be linked to the different kinds of inflammation found in different organs. A further investigation into the types of diseases and kinds inflammation might explain the sub-classification rationale and should be further investigated.

In this report, we made a very important discovery: a very high percentage of “Qing Re Yao” actually work on more than one inflammation pathway. This could be due to the poly-chemical nature of the herbs. The effect on the multiple pathways explains why some “Qing Re Yao” have been reported to have different effects on inflammation-associated diseases. For example, *Scutellaria baicalensis* has been shown to have multiple effects on iNOS, COX2, NFκB as well as inflammatory cytokines like IL-1β, IL-2, IL-6, IL-12 and TNF-α^23,24^ via decreasing the expression of IL-6 and the production of tumor necrosis factor alpha (TNF-α)^25,26^. It has distinct effects in the treatment of inflammatory disease. *Forsythia suspense* inhibits TNF-α and COX2 production^27,28^.

It is interesting to point out that “Qing Re Yao” developed from over a thousand years of human experience for the treatment of inflammation-related disease. Compared to the long history of Chinese medicine, the concept of combination therapy for the treatment of diseases has only recently become popular. From a therapeutic point of view, it seems acceptable that using a combinatorial approach of drugs to hit multiple targets could be a better way to control complex diseases. In contrast the “single drug and single target” approach may have limited value in controlling complicated diseases. In TCM formulations multiple “Qing Re Yao” herbs are used, supporting the notion that hitting multiple targets enhances their efficacy. Thus “Qing Re Yao” combination therapy has great synergistic potential that can further diversify its application for different diseases.

Another interesting finding is that different “Qing Re Yao” herbs could have different spectra and potency on downstream target genes (Table 1, Figs 3 and 4). This could be due to the complexity of the endogenous promoter on target gene expression. Combining different “Qing Re Yao” herbs into tailored “heat” removing formulations could broaden the inhibition dynamics of specific target genes, thereby creating a different inhibition spectrum for handling different diseases. The few “Qing Re Yao” cases,(herbs: 4, 13, 15 and 49), which did not show inhibition on our selected inflammation pathways may have actions on other inflammation signaling pathways, such as directly inhibiting virus or bacteria, or other STAT pathways. These herbs may even have synergistic enhancement effects on the “Qin Re Yao” combination.

It should be noted that “Qing Re Yao” is not the only category of herbs exhibiting anti-inflammation activity. Another category “Bu Yi Yao” did have a few herbs with anti-inflammation activity. For example, *Peoria Lactiflora* (herb 55) is used to treat painful or inflammatory disorders in traditional Chinese medicine. *Lycium barbarum* (herb 57) is used as a “cooling” agent for fever and night sweats in East Asian traditional medicine. *Paeonia Lactiflora* and *Lycium barbarum* have anti-inflammatory activity^29–31^. *Acanthopanax senticosus* (herb 56) is reported to have pharmacological action in the treatment of various diseases, such as chronic renal failure, rheumatics, diabetes mellitus, chronic bronchitis, hypertension, gastric ulcers and ischemic heart diseases^32–34^. 93% of “Qing Re Yao” affected one of the inflammatory pathways, compared to “Bu Yi Yao” herbs where a much lower 49% had anti-inflammatory effects. Most importantly only two herbs, (herbs 65, 78), in the ‘Bu Yi Yao” category exhibited activity on two anti-inflammation pathways and none demonstrated having more than three anti-inflammation properties. (Table 2). It is important to make the distinction that that TCM herbs also have a different classification based on their “energy” property (heat/warm, cold/cool, neutral) which is a different classification based on their usage such as “Qing Re Yao” or “Bu Yi Yao” or others. In “Bu Yi Yao”, 26 herbs have the “heat/warm” property, 11 herbs have the “cold or cool” property, and 10 herbs have the “neutral” property. There is no significant difference about affecting anti-inflammatory activity between 3 different “energy” property groups of “Bu Yi Yao” (Table S3). The “energy” property refers to the capacity to generate sensations and is different from the “heat” from inflammation being studied and is thus not taken into consideration of the multiple anti-inflammatory target effect. Other categories of herbs were also studied and actually showed much less frequency of poly-anti-inflammatory properties. In other words, the proportion of multiple anti-inflammatory activity in “Qing Re Yao” is significantly higher than any of other herb categories and unique. It should be pointed out other categories of herbs could also affect inflammation but have much less frequency in having poly inflammatory properties. The data is unpublished and not shown here. In summary, the biological activity nature of “Qing Re Yao” could be due to their poly anti-inflammatory activities. The other herbs even have one or two anti-inflammatory activities but could have other properties to distinguish themselves as a different category, such as “Bu Yi Yao”. Further studies of other categories of herbs are currently under investigation.

The results of the study provided valuable database information that can be mined for the active chemical(s) components. Once determined, these ingredients can be further developed into an even more potent anti-inflammation drug. One way to do this is by determining the intersection of different chemical components between different herbs and eliminating it in order to deduce the unique constituent compounds. Currently we are trying to identify the active compounds by comparing the biological and chemical fingerprints of each “Qing Re Yao” herb using biostatistics and bioinformatics technology. This will help provide an understanding of the science behind different inflammation mechanisms of “Qing Re Yao” herbs consequently leading to a new platform for drug discovery.

In this first study of an entire category of Chinese medicine herbs, we found that the majority of the “Qing Re Yao” herbs demonstrate anti-inflammatory activity and that each herb uses a combination of multiple mechanisms for targeting inflammation. Despite the fact that the TCM herbs in different categories exhibit anti-inflammatory and immune-modulatory activity, herbs categorized as “Qing Re Yao” share the unique quality that they can affect multiple anti-inflammation mechanisms at once compared with other herb classes. Though we did not test all anti-inflammation-related pathways and all the TLR-like activities, the proportion of herbs having multiple anti-inflammatory activity could be even higher. By studying these herbs, we propose a new paradigm for isolating effective anti-inflammatory compounds that can ultimately lead to new drug discoveries.

## Materials and Methods

226 kinds of herb granules were purchase from Guangdong E-fong Pharmaceutical in China or collected in our lab. “Qing Re Yao” and “Bu Yi Yao” list see Tables S1, S2. The Chinese herbs granules from E-fong pharmaceutical company were extracted by water and spray dried, the extract was prepared by first incubating 100 mg Chinese herbs granules in 1 ml sterile water at 80 °C for 30 minutes and then cooled down to room temperature in order to reconstitute. The preparation was then centrifuged at 12,000 rpm for 10 minutes to remove any soluble particles. The supernatant was used or stored at −20 °C until experiment use. β-glucuronidase is a major enzyme produced by the bacteria in the gastrointestinal tract. Side-chain sugar moieties are removed by β-glucuronidase. To subject the extract for *E.coli* β-glucuronidase treatment, the extract was diluted to 50 mg/ml with 100 mM of pH 6.8Tris-HCl buffer and then incubated with recombinant *E.coli* β-glucuronidase, 40 μg/ml at 37 °C for one hour.

HEK293 and MCF Cell lines were obtained from the American Type Culture Collection (ATCC) CRL-1573 and CRL-10782. HEK293 stable cell lines harboring NF-kB, STAT3, GAS response elements with luciferase reporter in pGL4 vector were made in the laboratory and maintained in DMEM containing 4.5 g/l glucose supplemented with 10% FBS. MCF7 stable cell line harboring GRE response elements with luciferase reporter in pGL4 vector was made in the laboratory and maintained in DMEM containing 4.5 g/l glucose supplemented with 10% dFBS. When GRE-MCF7cells were used for screening, they were maintained in DMEM containing 4.5 g/l glucose without PR supplemented with 2% cdFBS. All cell lines were maintained in a humidified incubator at 37 °C with an atmosphere of 95% air and 5% CO_2_.

Chemical fingerprints of all the herbs studied were done using LC-MS technology. The data is stored in the database of our laboratory and is accessible to the public upon request.

### Signaling Pathway Luciferase Reporter Assay

HEK-293 cell line stably harboring NFκB, STAT3, GAS response elements sequence and MCF7 cell line stably harboring GRE response element sequence in pGL4.0 luciferase vector (Promega) were used for the signaling pathway reporter assay. Cells were treated with 25 ng/ml TNF-α to stimulate NFκB signaling pathway, with 10 ng/ml IL-6 to stimulate STAT3 pathway, 0.5 µg/ml interferon gamma to stimulate GAS pathway and 300 nM Dexamethasone to stimulate GRE signaling pathway for 1 hour prior to the addition of herbs for another 6 to 12 hours. After the medium was removed at the end of the treatment, and cell extracts were prepared and luciferase activity was measured by Luciferase assay kit (Promega) according to the manufacturer’s instructions. IC_50_ was defined as the concentration of drug that inhibited stimulator-triggered luciferase activity by 50% after continuous drug exposure for 6–12 hours or stimulate GRE signal pathway.

### COX2 Activity Assay

Details of COX2 enzyme assay were published in our previous report^35^. COX2 (Cayman Chemical) enzymatic reactions were performed according to manufacturer’s instructions. The prostanoid product was quantified by LC-MS.

### iNOS Activity Assay

Details of iNOS enzyme assay were published in our previous report^35^. iNOS activity was measured by a colorimetric nitrite assay. The optical density was measured at 540 nm.

### Quantitative RT-PCR

HEK293 cells were seeded in 6-well plates treated with a different herbs extraction for 1 hour, then treated with 10 ng/ml IL-6 for an additional 6 hours. Total RNA was isolated using high pure RNA isolation kit (Roche, German). cDNA was synthesized using random primers and MMLV reverse transcriptase (New England Biolabs, Ipswich, MA). The real-time RT-PCR oligonucleotide primers used were: 5′-GCCACGGCTGCTTCCAGCTCC-3′ and 5′-TTGTGCTGGGTGCCAGGGCAGTGA-3′ forward and reverse primer for human β-actin, 5′-CTGGCTTTTGTGACGGAAAT-3′ and 5′-GTTTCCGGCACCTTCAGACT-3′forward and reverse primer for human BCL6; 5′-AGGCTCGGTTTCAGGAGTTT-3′; 5′-GAACAGCCCTTCTACCACGA-3′ forward and reverse primer for human JUNB; 5′-CCTTCCTCCTGTGCCTGATG-3′ and 5′-ACAATCTCATTTGAATCAGGAAGCT-3′ forward and reverse primer for human COX2; 5′-ACACATGGATCTAAGAGAAGAGG-3′ and 5′-CTATCAGGGAGCAGAGAAGG-3′ forward and reverse primer for human IL23. 5′-TTCACACAGGCCGATACAAAG-3′ and 5′-CATGGCACAGCGAAAGTTGG-3′ forward and reverse primer for human IRF1; 5′-GATACAGCTAAGACCATGTTCCG-3′ and 5′-GCCAGAGACGATTCCTGGT-3′ forward and reverse primer for human IRF9. 5′-AGGTACTGCTCTCCATCTAC-3′ and 5′-AGTGTAAGGGAGTCAACAGA-3′ forward and reverse primer for human TAP1; 5′-GCAGGCTGTACTATCTGCGAA-3′ and 5′-AGAGCCGAGTCCCATGTTCAT-3′ forward and reverse primer for human PSMB8; 5′-GTGGACGTTCGGGAGCTAC-3′ and 5′-ACTGGCCGATTTGGCACAG-3′ forward and reverse primer for human IFI35. The reaction was set up in duplicates in 15 µl total volumes with each primer and 7.5 µl IQ^TM^ SYBR Green Supermix (BioRad). The PCR cycle was as follows: 95 °C for 10 min, 40 cycles of 95 °C for 15 s, 60 °C for 1 min. Assays were performed using the iCycleriQ Real-Time Thermocycler Detection System (Bio-Rad). The fold increase or decrease was determined relative to a blank control after normalized.

Data analysis was presented as the mean ± SEM from at least three experiments. Inhibition was cut defined as more than 50% inhibition; stimulation was defined as more than 3 times stimulated vs the baseline of stimulator activity at concentration of herbs under 750 µg/ml in luciferase reporter signaling assay or under 4 mg/ml in enzyme assay. The normal control group (no herb-treated group) was used as the baseline. Data was analyzed by one-way or two-way ANOVA (GraphPad Prism 6), Student’s T-test (Microsoft Office Excel). The difference was considered to be statistically significant when P < 0.05.

## Electronic supplementary material

Dataset 1, Dataset 2 and Dataset 3
