# Supplementary material for: Majority of Chinese Medicine Herb Category “Qing Re Yao” Have Multiple Mechanisms of Anti-inflammatory Activity
Source: Sci Rep. 2018 May 9;8:7416. doi: 10.1038/s41598-018-25813-x (PMC5943244; doi:10.1038/s41598-018-25813-x)
Supplement: Supplementary file 1 — Dataset 1, Dataset 2 and Dataset 3 [file 41598_2018_25813_MOESM1_ESM.doc]

**Majority of Chinese Medicine Herb category “Qing Re Yao” Have Multiple Mechanisms of Anti-inflammatory Activity**

Fulan Guan^#^, Wing Lam^#^, Rong Hu, Yun Kyung Kim, Hua Han and Yung-Chi Cheng*

Department of Pharmacology

Yale University School of Medicine, New Haven, Connecticut, 06510, USA

^#^ These authors contributed equally to this work

Correspondence and requests for materials should be addressed to Yung-Chi Cheng

Telephone number: 2037857119

Email: [yccheng@yale.edu](mailto:yccheng@yale.edu)

Address: 333 Cedar Street, Yale University School of Medicine, Department of Pharmacology, SHM B254, New Haven, Connecticut 06510, USA

**Supplementary Materials**

**Table S1-S2**

**Table S1. List of Qing Re Yao**

| Latin Name | English Name |
| --- | --- |
| Hedyotis diffusa | Spreading Hedyotis Herb |
| Pulsatilla chinensis | Chinese Pulsatilla Root |
| Lobelia chinensis | Chinese Lobelia Herb |
| Isatis indigotica | Isatis Root |
| Scutellaria barbata | Barbated Skullcup Herb |
| Andrographis paniculata | Common Andrographis Herb |
| Paris polyphylla | Paris Rhizome |
| Rehmannia glutinosa | Rehmannia Root |
| Dryopteris crassirhizoma | Male Fern Rhizome |
| Phellodendron chinense | Amur Cork-tree |
| Coptis chinensis | Golden Thread |
| Scutellaria baicalensis | Baical Skullcap Root |
| Gentiana manshurica | Chinese Gentian |
| Lasiosphaera fenzlii | Puff-ball |
| Portulaca oleracea | Purslane Herb |
| Taraxacum mongolicum | Dandelion |
| Isatis indigotica | Natural Indigo |
| Artemisia annua | Sweet Wormwood Herb |
| Celosia argentea | Feather Cockscomb Seed |
| Lonicera japonica | Honeysuckle Stem |
| Lonicera japonica | Honeysuckle Flower |
| Sophora flavescens | Lightyellow Sophora Root |
| Forsythia suspensa | Weeping Forsythia Capsule |
| Oroxylum indicum | Indian Trumpetflower Seed |
| Belamcanda chinensis | Blackberrylily Rhizome |
| Mahonia bealei | Leatherleaf Mahonia Leaf |
| Houttuynia cordata | Heartleaf Houttuynia Herb |
| Trichosanthes kirilowii | Snakegourd Root |
| Scrophularia ningpoensis | Figwort Root |
| Anemarrhena asphodeloides | Common Anemarrhena Rhizome |
| Viola yedoensis | Tokyo Violet Herb |
| Chrysanthemum indicum | Wild Chrysanthemum Flower |
| Cassia obtusifolia | Cassia Seed |
| Paeonia suffruticosa | Tree Peony Bark |
| Lycium barbarum | Chinese Wolfberry Root-bark |
| Sargentodoxa cuneata | Sargentgloryvine Stem |
| Patrinia scabiosaefolia | Dahurian Patrinia Herb with Root |
| Euphorbia humifusa | Creeping Euphorbia |
| Fraxinus rhynchophylla | Ash Bark |
| Isatis indigotica | Dyers Woad Leaf |
| Phragmites communis | Reed Rhizome |
| Lithospermum erythrorhizon | Arnebia Root |
| Gardenia jasminoides | Cape Jasmine Fruit |
| Sophora tonkinensis | Vietnamese Sophora Root |
| Cremastra appendiculata | Appendiculate Cremastra Pseudobulb |
| Cynanchum atratum | Blackend Swallowwort Root |
| Picrorhiza scrophulariiflora | Figwortflower Picrorhiza Rhizome |
| Prunella vulgaris | Common Selfheal Fruit-Spike |
| Stellaria dichotoma | Starwort Root |
| Dictamnus dasycarpus | Densefruit Pittany Root-bark |
| Paeonia veitchii | Red Peony Root |
| Buddleja officinalis | Pale Butterflybush Flower |
| Eriocaulon buergerianum | Pipewort Flower |
| Geranium wilfordii Maxim | Herba Geranii |

Table S2. List of Bu Yi Yao

| Latin Name | English Name |
| --- | --- |
| Paeonia lactiflora | White Peony Root |
| Glehnia littoralis | Coastal Glehnia Root |
| Acanthopanax senticosus | Manyprickle Acanthopanax Root |
| Lycium barbarum | Barbary Wolfberry Fruit |
| Sesamum indicum | Black Sesame |
| Panax ginseng | Red Ginseng |
| Astragalus membranaceus | Milkvetch Root |
| Polygonum multiflorum | Prepared Fleeceflower Root |
| Eucommia ulmoides | Eucommia Bark |
| Nelumbo nucifera | Lotus Seed |
| Allium tuberosum | Tuber Onion Seed |
| Cynomorium songaricum | Songaria Cynomorium Herb |
| Pseudostellaria heterophylla | Heterophylly Falsestarwort Root |
| Eclipta prostrata | Yerbadetajo Herb |
| Adenophora stricta | Fourleaf Ladybell Root |
| Morus alba | Mulberry Fruit |
| Astragalus complanatus | Flatstem Milkvetch Seed |
| Dendrobium loddigesii | Dendrobium |
| Cuscuta chinensis | Dodder Seed |
| Dipsacus asperoides | Himalayan Teasel Root |
| Lilium lancifolium | Lily Bulb |
| Ophiopogon japonicus | Dwarf Lilyturf Tuber |
| Morinda officinalis | Morinda Root |
| Psoralea corylifolia | Malaytea Scurfpea Fruit |
| Dioscorea opposita | Common Yam Rhizome |
| Cistanche deserticola | Desertliving Cistanche |
| Angelica sinensis | Chinese Angelica |
| Rehmannia glutinosa | Prepared Rehmannia Root |
| Dimocarpus longan | Longan Aril |
| Asparagus cochinchinensis | Cochinchinese Asparagus Root |
| Polygonatum kingianum | Solomonseal Rhizome (processed) |
| Ligustrum lucidum | Glossy Privet Fruit(processed) |
| Atractylodes Macrocephala | Largehead Atractylodes Rhizome |
| Dolichos lablab | White Hyacinth Bean |
| Curculigo orchioides | Common Curculigo Rhizome |
| Trigonella foenum-graecum | Common Fenugreek Seed |
| Alpinia oxyphylla | Sharpleaf Glangal Seed |
| Gekko gecko | Giant Gecko |
| Epimedium brevicornum | Epimedium Herb |
| Panax quinquefolium | American Ginseng |
| Panax ginseng | Korea Ginseng |
| Glycyrrhiza uralensis | Liquorice Root |
| Eurylae ferox | Gordon Euryale Seed |
| Polygonatum odoratum | Fragrant Solomonseal Rhizome |
| Cordyceps militaris | Cordyceps militaris |
| Ganoderma lucidum (Leyss.ex Fr.) Karst. | Ganoderma lucidum |
| Ziziphus zizyphus | Jujube |

Table S3 The Percentage of different property of “Bu Yi Yao” in affecting on the inflammatory cytokine or glucocorticoid signaling pathway and COX2, iNOS enzyme activity.

| **Bu Yi Yao** | **iNOS** | **COX2** | **INF-γ** | **IL-6** | **TNF-α** | **Dexa** | **Any one of them** | **Any 2 or more of them** | **Any 3 or more of them** |
| --- | --- | --- | --- | --- | --- | --- | --- | --- | --- |
| **total(47)** | **34%** | **13%** | **2%** | **0%** | **0%** | **4%** | **49%** | **4%** | **0%** |
| **"heat" property(26)** | 38% | 19% | 4% | **0%** | **0%** | 4% | 54% | 7% | **0%** |
| **"cold" property(11)** | 45% | 0% | 0% | **0%** | **0%** | 0% | 45% | 0% | **0%** |
| **Neutral property(10)** | 10% | 10% | 0% | **0%** | **0%** | 10% | 30% | 0% | **0%** |

TNF-α: TNFα-NFκB luciferase reporter; IL-6: IL-6-STAT3 luciferase reporter; INF-γ: INFγ-GAS luciferase reporter; Dexa: Dexamethasone-GRE luciferase reporter; COX2: COX2 enzyme activity; iNOS: iNOS enzyme activity.
